# A Good Cough Mixture

**Published:** 1891-10

**Authors:** 


### A Good Cough Mixture.

℞. Acidi hydrocyanici dil..................

Chloroformi purif, aa....................xxxvjii minims.

Tincturœ hyoscyami......................

Syrupi tolutani.............................

Aquæ camphoræ, aa........................vj fluid drachms.

Mucilagonis acaciæ.........................v fluid drachms.

M. Sig. One tablespoonful every two to four hours. For children in proportionate doses.

